# A Protocol for Improved Precision and Increased Confidence in Nanoparticle Tracking Analysis Concentration Measurements between 50 and 120 nm in Biological Fluids

**DOI:** 10.3389/fcvm.2017.00068

**Published:** 2017-11-03

**Authors:** Martin E. M. Parsons, Damien McParland, Paulina B. Szklanna, Matthew Ho Zhi Guang, Karen O’Connell, Hugh D. O’Connor, Christopher McGuigan, Fionnuala Ní Áinle, Amanda McCann, Patricia B. Maguire

**Affiliations:** ^1^SPHERE Research Group, UCD Conway Institute, University College Dublin (UCD), Dublin, Ireland; ^2^School of Biomolecular and Biomedical Science, University College Dublin (UCD), Dublin, Ireland; ^3^School of Mathematics and Statistics, University College Dublin (UCD), Dublin, Ireland; ^4^School of Medicine, College of Health and Agricultural Science (CHAS), University College Dublin (UCD), Dublin, Ireland; ^5^UCD Conway Institute of Biomolecular and Biomedical Science, University College Dublin (UCD), Dublin, Ireland; ^6^Department of Neurology, St. Vincent’s University Hospital, Dublin, Ireland; ^7^Department of Obstetrics, Rotunda Hospital, Dublin, Ireland; ^8^Department of Haematology, Mater Misericordiae University Hospital, Dublin, Ireland; ^9^UCD Institute for Discovery, O’Brien Centre for Science, University College Dublin (UCD), Dublin, Ireland

**Keywords:** nanoparticle tracking analysis, extracellular vesicles, plasma, serum, platelets, platelet releasate, particle enumeration

## Abstract

Nanoparticle tracking analysis (NTA) can be used to quantitate extracellular vesicles (EVs) in biological samples and is widely considered a useful diagnostic tool to detect disease. However, accurately profiling EVs can be challenging due to their small size and heterogeneity. Here, we aimed to provide a protocol to facilitate high-precision particle quantitation by NTA in plasma, the supernatant of activated purified platelets [the platelet releasate (PR)] and in serum, to increase confidence in NTA particle enumeration. The overall variance and the precision of NTA measurements were quantified by root mean square error and relative standard error. Using a bootstrapping approach, we found that increasing video replicates from 5 s × 60 s to 25 s × 60 s captures led to a reduction in overall variance and a reproducible increase in the precision of NTA particle-concentration quantitation for all three biofluids. We then validated our approach in an extended cohort of 32 healthy donors. Our results indicate that for vesicles sized between 50 and 120 nm, the precision of routine NTA measurements in serum, plasma, and PR can be significantly improved by increasing the number of video replicates captured. Our protocol provides a common platform to statistical compare particle size distribution profiles in the exosomal-vesicle size range across a variety of biofluids and in both healthy donor and patient groups.

## Introduction

Extracellular vesicles (EVs) are released by nearly all eukaryotic cells and are found in a diverse range of human biofluids. They regulate a diverse range of biologic and inflammatory pathologic processes and have been implicated in myriad of diseases (1–3). As such, they have emerged as a promising source of future biomarkers in biofluids with both diagnostic and prognostic value (4, 5). However, accurately profiling EVs can be challenging due to their small size and heterogeneity (6). While important advances have been made (5), optimization of procedures for EV quantification across laboratories is of great significance to the EV field with implications for both basic and clinical research.

Nanoparticle tracking analysis (NTA) is widely used to determine the particle size distribution of a sample (6–9). Particle size distribution describes the concentration of particles as a function of size (10). In brief, particle size is determined by focusing a laser beam through a suspension of particles. The light scattered by individual particles in solution allows visualization of particles and each individual particles’ displacement is recorded over disjointed time intervals (8, 11, 12). The mean square displacement for each particle is then used, alongside liquid temperature and viscosity, to calculate individual particle sizes using the Stokes-Einstein equation. The concentration of particles is determined by counting total particles and representing the concentration as a binned histogram (13).

The validity of particle size distributions for a sample depends on accurate sizing of particles as well as precise concentration measurements. Accuracy is generally within 5% of the expected particle size once correct hardware and software setting have been applied (7, 14, 15). However, NTA concentration measurements have been shown to have low precision, due to variation in the number of particles detected between video replicate measurements of the same sample (12). For low particle counts, it has been suggested that increasing video replicates could lead to improved concentration measurements (7).

Here, we sought to determine the effect of increasing video replicates on the precision of particle concentration quantitation using NTA in the biofluids of plasma, serum, and the supernatant of activated purified platelets [the platelet releasate (PR)]. Although plasma and serum are widely used in clinical diagnoses (2, 16, 17), the PR is an important biofluid to also include as contains a variety of EVs (18–20) and its contents play a fundamental role in hemostasis, wound healing, and the inflammatory response (21–25). In our analysis, we initially used a bootstrapping approach to investigate the precision of particle concentration measurements in all three biofluids. We found that increasing video replicates led to a reproducible increase in the precision of NTA particle concentration quantitation in these biofluids and we validated our findings in an extended clinical cohort.

## Materials and Methods

### Plasma, PR, and Serum Isolation

Human plasma, serum, and platelets were obtained from healthy adult volunteers in accordance with approved guidelines from the UCD research, and with ethical approval from St. Vincent’s University Hospital and the Rotunda Hospital Research Ethics committees. All subjects gave their informed written consent according to the declaration of Helsinki.

Isolation of platelet-free plasma and PR was as described (19). Briefly, 44 ml of blood was drawn into acid citrate dextrose blood collection tubes (BD, Franklin Lakes, NJ, USA) and the first 4 ml was discarded. Blood was centrifuged at 150 × *g* for 10 min at room temperature and platelet-rich plasma was aspirated. Platelets were pelleted from platelet rich plasma by centrifugation at 720 × *g* for 10 min at room temperature and platelet poor plasma was aspirated. Platelets were suspended in a modified Tyrode’s buffer (130 mM NaCl, 10 mM trisodium citrate, 9 mM NaHCO_3_, 6 mM dextrose, 0.9 mM MgCl_2_, 0.81 mM KH_2_PO_4_, 10 mM Tris pH 7.4). Platelet count was adjusted to 1 × 10^9^/ml and platelets were stimulated with 1 U thrombin/ml at 37°C in a PAP-4 aggregometer under constant stirring. The supernatant from the thrombin activated platelets was centrifuged three times at 10,000 × *g* for 10 min at 4°C to remove the aggregated platelets and cell debris, leaving the activated platelet supernatant, or PR.

Serum was prepared by drawing 4 ml whole blood into serum blood collection tubes (BD, Franklin Lakes, NJ, USA) and processed according to manufacturer’s instructions. In brief, samples were rested upright for 60 min to allow RBCs to clot. The RBC clot was subsequently pelleted by centrifugation at 1,300 × *g* for 10 min and serum was aspirated. All plasma, serum, and PR samples were stored at −80°C prior to NTA analysis.

### NTA of Biological Samples

Particle size distribution in PR, plasma, and serum samples was determined by NTA using a NanoSight NS300 system (Malvern Technologies, Malvern, UK) configured with a 488 nm laser and a high sensitivity scientific CMOS camera. Samples were diluted (PR 1:20–1:50, plasma 1:100–1:2,000, serum 1:500) in particle-free PBS (Gibco, Waltham, MA, USA) to an acceptable concentration, according to the manufacturers recommendations. Samples were analyzed under constant flow conditions (flow rate = 50) at 25°C according to Ref. (7, 26). For bootstrapped samples, 30 s × 60 s successive videos were captured with a camera level of 16. Data were analyzed using NTA 3.1.54 software with a detection threshold of 5. For the validation cohort, 15 s × 60 s videos were captured with a camera level of 16 and a detection threshold of 10.

### Statistical Analysis of Nanoparticle Analysis Tracking Data

Particle size distributions were binned into 10 nm bin widths using NTA 3.1.54 software for all video replicates to determine concentration measurements. To understand the variability in the estimates across the complete range of bin widths, we quantified the root mean square error (RMSE) of 100 bootstrap samples of *n* = 5, 10, 15, 20, and 25 NTA recordings, which were sampled from the available 30 concentration measurements. The RMSE is the error sum of squares scaled to the data from which it was derived and was calculated as follows:

$${\mathrm{RSME}}_{m}=\;\sqrt{\frac{\sum_{i=1}^{N_{\mathrm{bins}}}\sum_{j=1}^{N_{\mathrm{boot}}}{\left(x_{ij}-\;{\bar{x}}_{i}\right)}^{2}}{N_{\mathrm{bin}}\left(N_{\mathrm{boot}}-1\right)}}$$

where *N*_bins_ is the number of bins considered, *N*_boot_ is the number of bootstrap samples taken for each bin, *x*_ij_ is the *j^th^* bootstrap mean for bin *i* and ${\bar{x}}_{i}$ is the mean of the bootstrap means for bin *i*.

Next, to determine the precision of the concentration estimates, we quantified the relative standard error (RSE) as follows:

$${\mathrm{RSE}}_{n}=\;\frac{\frac{s}{\sqrt{n}}}{{\bar{c}}_{n}}\;\times 100=\;\frac{s}{{\bar{c}}_{n}\sqrt{n}}\;\times 100$$

where *s* is the SD of measurements, ${\bar{c}}_{n}$ is the sample mean concentration estimate, and *n* is the number of samples. Precision of our clinical cohort was quantified by RSE where *n* = 5 or 15 video replicates. Initially, 100 bootstrap samples were sampled from the available 30 concentration measurements and the RSE was calculated for each bin width for n = 5, 10, 15, 20, and 25 video replicates. In our validation cohort, RSE was calculated from *n* = 5 and *n* = 15 video replicates for each bin width.

### Statistical Analysis

All bootstrapping, RMSE, and RSE calculations were performed using the open source programming language for statistical computing R 3.3.1 (27) and the integrated development environment RStudio 1.0.136 (28).

## Results

### Reduction in the Overall Variance of NTA Measurement by Increasing Video Replicates

Using a bootstrapping approach, we examined the effect of increasing video replicates on the total variance of NTA measurement of particles in plasma, PR, and serum. RMSE, a measure of total variance, was calculated to show the variability of measurement at each time point. Plasma, PR, and serum were isolated from three healthy donors and subjected to analysis by NTA. 30 s × 60s consecutive videos were captured for each sample. All experiments were performed in triplicate. RMSE was calculated for 100 bootstrap samples of *n* = 5, 10, 15, 20, and 25 video replicates. RMSE was normalized to *n* = 5 video replicates with *n* = 5 representing 100% variance. For plasma, PR, and serum increasing video replicates led to a decrease in total variance across all samples (Table 1). On average, doubling the number of video replicates from *n* = 5 to *n* = 10 led to a ~ 30% reduction in the initial variance, with further reductions in total variance reproducibly replicated in all samples with increasing video captures.

**Table 1:** Overall variance of nanoparticle tracking analysis measurements shows an exponential decay in variance as *n* number bootstrapped video replicates increase.

	Root mean square error (RMSE) represented as % of *n* = 5 video replicates (±SD)		
*n* number	Plasma	Platelet releasate	Serum
5	100	100	100
10	70.4 ± 4.27	70.4 ± 1.88	70.2 ± 5.33
15	57.6 ± 3.81	58.0 ± 3.58	58.0 ± 4.93
20	50.5 ± 4.24	49.9 ± 3.26	51.4 ± 3.98
25	44.5 ± 3.32	45.2 ± 2.28	45.4 ± 2.82

*Average RMSE (±SD) for each plasma, PR and serum is represented as a percentage of RMSE at 5 min. A consistent % reduction in RMSE observed for each sample with increased *n* bootstrapped samples*.

### Precision of NTA Concentration Measurements in Individual Bin Widths Is Improved with Increasing the Number of Video Captures

**Figure 1:**
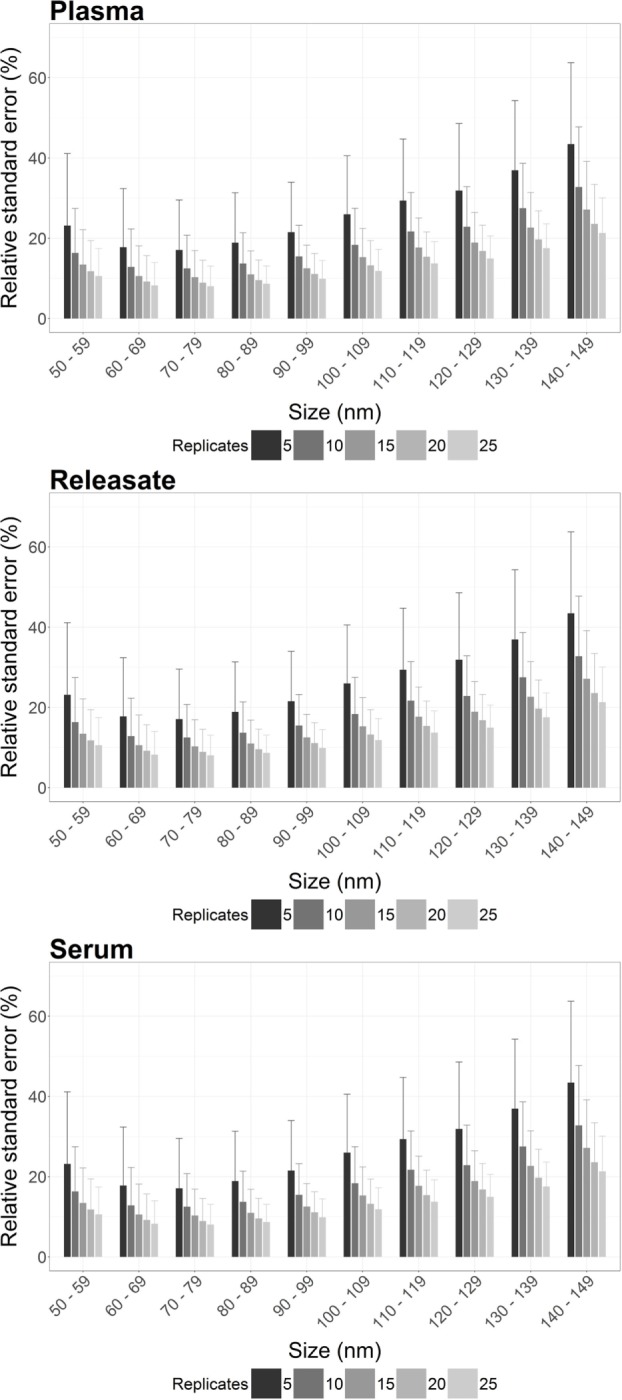
The precision of nanoparticle tracking analysis (NTA) concentration measurements is enhanced by increasing the number of video replicates in all bin widths. For plasma, platelet releasate and serum the average relative standard error (RSE) per bin width from a total of 900 bootstrapped samples was reproducibly decreased by increasing *n* video replicates. As RSE is a measure of the statistical precision of a sample measurement, the reduction in RSE with increased video replicates indicated that more video replicates led to increased precision of NTA measurements.

Individual RSE was calculated per 10 nm bin width for 100 bootstrap samples from plasma, PR, and serum, for *n* = 5, 10, 15, 20, and 25 video replicates. A reduction in RSE was reproducibly observed across all bin widths for plasma, PR, and serum with increasing video replicates (Figure 1; Table S1 in Supplementary Material).

However, also of interest is the maximum RSE across all bootstrapped samples. Focusing on particles from 100 to 110 nm, the maximum RSE was found to be excessively high for *n* = 5 replicates only, with a maximum RSE for plasma of 95.3, 49.2% for PR, and 63.1% for serum (Table 2). However, increasing *n* video replicates had a reproducible significant reduction in maximum RSE. For example, for *n* = 10 video replicates, maximum RSE for plasma was 52.8, 25.8% for PR, and 26.6% for serum. Further reductions in maximum RSE were evident with increased *n* video replicates (Table 2). This pattern was reproduced for all bin widths. The maximal RSE for all bin widths and samples can be found in Table S1 in Supplementary Material. As RSE is a measure of the precision of concentration measurements by NTA, an overall reduction in maximal RSE indicates that increasing video replicates leads to a reproducible increase in the precision of particle concentration measurements by NTA.

**Table 2:** The precision of nanoparticle tracking analysis concentration measurement at 100–110 nm is increased by increasing the number of video replicates.

	Plasma	Platelet releasate	Serum
	
*n* number	Maximum relative standard error (RSE) (%)	Maximum RSE (%)	Maximum RSE (%)
5	95.3	49.2	63.1
10	52.8	25.8	26.6
15	41.0	21.2	20.2
20	31.7	15.5	17.4
25	27.6	14.7	14.2

*Focusing on particles from 100 to 110 nm, the maximum RSE is significantly decreased by increasing video replicates*.

### Validation of Increased Precision of NTA Concentration Measurements in a Larger Cohort of 32 Donors

**Figure 2:**
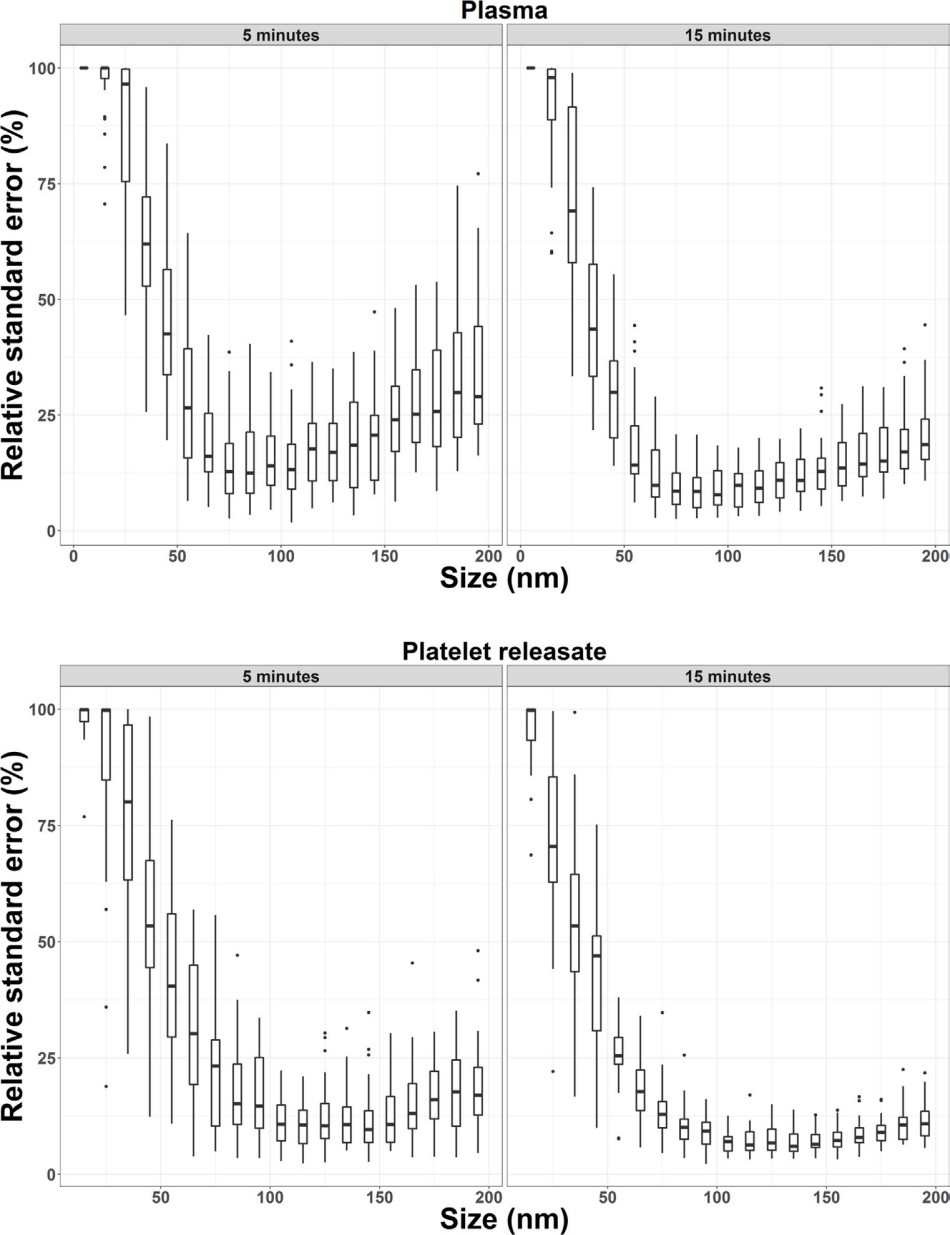
Precision of nanoparticle tracking analysis concentration measurements from 32 healthy donors is increased by increasing the number of video replicates in all bin widths. For plasma and platelet releasate, the average relative standard error per bin width from 32 healthy donors was reproducibly reduced by increasing video replicates from *n* = 5 to *n* = 15.

Next, we wished to determine if the increase in precision of concentration quantitation observed in our bootstrapped samples could be replicated in a larger cohort (*n* = 32). 15 s × 60s NTA video replicates were captured for PR and plasma from 32 healthy donors. The first five videos were analyzed to determine the RSE of *n* = 5 video replicates for every 10 nm bin width for each donor. These five, plus an additional 10 videos were then analyzed to determine the RSE of *n* = 15 video replicates. By increasing the video replicates from 5 to 15, the precision of NTA concentration quantitation was improved (Figure 2; Table 3). The maximum RSE in plasma was reduced from 64.37 to 44.34%, while the maximum RSE in PR was reduced from 76.23 to 38.01%. In accordance with the findings from our bootstrapped samples, the precision was most improved in the bin centers corresponding to vesicles of 50–120 nm in size. For example, taking the bin center from 100 to 110 nm for illustrative purposes, the maximum RSE was found to be high with *n* = 5 replicates with maximum RSE for plasma of 41.0%, and 22.3% for PR (Table 4). Increasing *n* video replicates had a reproducible significant effect on maximum RSE. When video replicates were to *n* = 15, maximum RSE was 18.0 and 12.6% for plasma and PR, respectively, indicating a greatly improved precision of NTA concentration measurements (Table 4). In a similar manner, the maximum error was also calculated for each bin width across all samples (Table S2 in Supplementary Material).

**Table 3:** Increased precision of nanoparticle tracking analysis concentration measurements in a larger cohort of 32 healthy donors of particles from 50 to 120 nm.

	Plasma	Platelet releasate
	
*n* number	Maximum relative standard error (RSE) (%)	Maximum RSE (%)
5	64.37	76.23
15	44.34	38.01

**Table 4:** Precision of nanoparticle tracking analysis concentration measurements is increased in particles from 100 to 110 nm by increasing the number of video replicates in a cohort of 32 healthy donors.

		Plasma		Platelet releasate	
*n* number	Size (nm)	Average relative standard error (RSE) (%)	Maximum RSE (%)	Average RSE (%)	Maximum RSE (%)
5	100–110	15.6 ± 15.6	41.0	11.5 ± 11.5	22.3
15	100–110	9.3 ± 9.3	18.0	6.8 ± 6.8	12.6

*For particles from 100 to 110 nm, average RSE (±SD) is decreased by increasing the number of video replicates. Maximum RSE is also significantly decreased by increasing the number video replicates*.

## Discussion

Here, we sought to determine the effect of increasing video replicates on the precision of EV concentration quantitation using NTA. Initially, we used a bootstrapping approach to investigate the precision of particle concentration measurements in plasma, the PR, and in serum. Our results indicated that the precision of routine NTA measurements can be significantly improved in the particle size range of 50–120 nm for all biofluids analyzed and this held true even as we extended our analysis to plasma and PR in a larger cohort of 32 donors.

As the NTA method is the current gold standard to measure the size and concentration of small particles in biological samples and has the potential to be a useful diagnostic tool to detect disease (8, 29), our protocol has much relevance to the field. In fact, with NTA-acquisition settings constant between analyses, our approach will enable the mean, mode, and median particle size together with EV concentration to be more precisely compared between differential samples. In this way, we conclude that we now provide a common platform to statistically compare particle size distribution profiles from the plasma and PR of patients with a variety of pathologies.

## Ethics Statement

Human plasma, serum and platelets were obtained from healthy adult volunteers in accordance with approved guidelines from the UCD research ethics committee, and with ethical approval from St Vincent’s University Hospital and the Rotunda Hospital Research Ethics committees. All subjects gave their informed written consent according to the declaration of Helsinki.

## Author Contributions

MP, DM, PS, MG, AM, and PM designed research; MP, DM, PS, MG, KO, HO, CM, FÁ, AM, and PM performed experiments; MP, DM, PS, MG, AM, and PM analyzed data; MP, DM, PS, MG, KO, HO, CM, FÁ, AM, and PM wrote the paper.

## Conflict of Interest Statement

The authors declare that the research was conducted in the absence of any commercial or financial relationships that could be construed as a potential conflict of interest.

## Supplementary Material

Table S1 **Relative standard error per bin width from bootstrap samples**. 30 x 60 second consecutive videos were captured for each sample, plasma, platelet releasate (PR), and serum from 3 donors in triplicate. RSE was calculated for 100 bootstrap samples of n = 5, 10, 15, 20, and 25 video replicates. Average RSE per n video replicates for each bin size was calculated for all 900 bootstrapped samples. A reduction in RSE was reproducibly observed across all bin widths for plasma, platelet releasate (PR) and serum with increasing video replicates.

Table S2 **Relative standard error per bin with from 32 donor validation**. 15 x 60 second consecutive videos were captured for each sample, plasma and platelet releasate (PR) for 32 healthy donors. The first five videos were analysed to determine the RSE of n = 5 video replicates for every 10 nm bin width for each donor. These five, plus an additional 10 videos were then analysed to determine the RSE of n = 15 video replicates. The average RSE for each bin size for 5 and 15 minutes shows a reduction for each bin size. The maximum RSE for was reduced for each bin size for both biological samples.

## Abbreviations

EVs, extracellular vesicles; FTLA, finite track length adjustment; NTA, nanoparticle tracking analysis; PR, platelet releasate; RMSE, root mean square error; RSE, relative standard error.
